# Muito Mais que Apenas Mulheres: Mulheres Maravilha

**DOI:** 10.36660/abc.20220443

**Published:** 2022-07-29

**Authors:** Marcia Koike, Luciana Aikawa

**Affiliations:** 1 Universidade de São Paulo Faculdade de Medicina Departamento de Clínica Médica São Paulo SP Brasil LIM-51 - Laboratório de Investigação médica da Disciplina de Emergências Clínicas - Departamento de Clínica Médica, Faculdade de Medicina da Universidade de São Paulo (FMUSP), São Paulo, SP – Brasil; 2 Instituto de Assistência Médica do Servidor Público Estadual Pós-Graduação em Ciências da Saúde São Paulo SP Brasil Pós-Graduação em Ciências da Saúde, Instituto de Assistência Médica do Servidor Público Estadual (IAMSPE), São Paulo, SP – Brasil; 3 Hospital do Servidor Público Municipal Ambulatório de Acupuntura São Paulo SP Brasil Ambulatório de Acupuntura, Hospital do Servidor Público Municipal (HSPM), São Paulo, SP – Brasil

**Keywords:** COVID-19, SARS-CoV2, Pandemia, Betacoronavírus, Médicas, Ocupações em Saúde, Mulheres/psicologia, Prática Profissional, Profissional Médico, Eficiência, Desenvolvimento Pessoal/ética, Estilo de Vida, Epidemiologia

O impacto da pandemia de SARS-Cov2 atingiu o mundo de forma rápida e avassaladora, e colocou em evidência os heróis de capa branca: os profissionais da saúde. Foram mais de dois anos de trabalhos ininterruptos, enfrentando diversos desafios e conflitos externos e internos para preservar a vida do paciente, dos seus colegas, de seus familiares e a própria. Um cenário complicado, não somente pela sobrecarga de demanda profissional, mas também pelo desconhecimento cientifico do que estávamos enfrentando. Cientistas de todo mundo somaram suas forças para divulgação das evidências cientificas e, em paralelo, as sociedades médicas se mobilizaram para otimizar procedimentos de atendimentos com proteção ao profissional atuante.^1^

O estresse e a síndrome de Burnout fazem parte das doenças ocupacionais em profissionais de saúde.^2^ No entanto, o cenário peculiar da Covid-19 mostrou que apesar de não ser fácil estar na linha de frente no combate ao SARS-CoV2, pelo grau elevado de estresse e exaustão em enfermeiros e médicos demonstrado em estudos, as estratégias comportamentais individuais e sociais/coletivas podem ajudar.^3–6^ Este minieditorial é dedicado às médicas.

Elas que, além dos desafios nos papéis de mulher, mãe, filha, amiga, companheira, dona de casa, e tantos outros, também se esforçaram em jornadas exaustivas para cuidado do paciente. No estudo de Oliveira et al.,^7^ *a*s mulheres, que têm historicamente dupla jornada de trabalho, viram-se em Burnout durante a Pandemia de SARS-Cov2 no Brasil. Foram muitas demandas acumuladas, como jornadas de trabalho em dois ou três locais diferentes, afazeres domésticos, pouco tempo de lazer e perda salarial, mas se empenharam também em se manter criativas e manter sua boa qualidade de vida. Mais que médicas, verdadeiras Mulheres Maravilha, buscaram na espiritualidade, o conforto, a segurança e a redução do estresse.

A pandemia trouxe à tona desafios que requerem reflexão sobre nosso estilo de vida e como eles impactam na qualidade de vida. As médicas demonstram bravura, coragem e determinação, mas carecem ainda de mais cuidados com o bem-estar. As estratégias de enfrentamento de situações difíceis e cultivo de bem-estar devem ser implementadas nos centros médicos para que seus colaboradores possam praticá-las e vivenciá-las. Além dos exercícios físicos e dieta equilibrada, as ferramentas comportamentais como cultivo de pensamentos positivos e o sentimento de gratidão, praticar as diversas formas de meditação, Yoga, Tai Chi ou outras práticas similares pela natureza podem também aquietar a mente e reduzir o estresse.^8–10^
