# Supplementary material for: Muito Mais que Apenas Mulheres: Mulheres Maravilha
Source: Arq Bras Cardiol. 2022 Jul 29;119(2):317–8. [Article in Portuguese] doi: 10.36660/abc.20220443 (PMC9363059; doi:10.36660/abc.20220443)
Supplement: Translation

# Translation: Much More Than Just Women: Wonder Women

**Authors:** Marcia Koike, Luciana Aikawa

**Affiliations:** 1 São Paulo SP Brazil LIM-51 - Laboratório de Investigação médica da Disciplina de Emergências Clínicas - Departamento de Clínica Médica, Faculdade de Medicina da Universidade de São Paulo (FMUSP), São Paulo, SP – Brazil; 2 São Paulo SP Brazil Pós-Graduação em Ciências da Saúde, Instituto de Assistência Médica do Servidor Público Estadual (IAMSPE), São Paulo, SP – Brazil; 3 São Paulo SP Brazil Ambulatório de Acupuntura, Hospital do Servidor Público Municipal (HSPM), São Paulo, SP – Brazil

**Keywords:** COVID-19, SARS-CoV2, Pandemics, Betacoronavirus, Medicals, Health Occupations, Women/psychology, Professional Practice, Medical Profissional, Efficiency, Staff Development/ethics, Life Style, Epidemiology

The impact of the SARS-Cov2 pandemic hit the world quickly and overwhelmingly and highlighted the heroes of the white cover: healthcare professionals. It took more than two years of uninterrupted work, facing various challenges and external and internal conflicts to preserve the patient’s life, colleagues, their families and themselves. A complicated scenario, not only due to the overload of professional demand but also the scientific lack of knowledge of what we were facing. Scientists worldwide have joined forces to disseminate scientific evidence, and, at the same time, medical societies have mobilized to optimize care procedures with protection for active professionals.^1^

Stress and Burnout syndrome are part of occupational diseases in health professionals.^2^ However, the particular scenario of Covid-19 has shown that although it is not easy to be on the front lines in the fight against SARS-CoV2, nurses and doctors demonstrated high stress and exhaustion in studies, so behavioral/collective strategies can help.^3–6^ This mini-editorial is dedicated to female doctors.

In addition to the challenges in the roles of woman, mother, daughter, friend, companion, housewife and many others, they also struggled in exhausting journeys to take care of the patient. In the study by Oliveira et al.,^7^ women, who historically have a double shift, found themselves in Burnout during the SARS-cov2 Pandemic in Brazil. There were many accumulated demands, such as working hours in two or three different places, household chores, little leisure time and loss of salary, but they also struggled to maintain creativity and maintain a good quality of life. More than doctors, true Wonder Women sought spirituality, comfort, safety and stress reduction.

The pandemic has brought to light challenges that require reflection on our lifestyle and how they impact our quality of life. Doctors demonstrate bravery, courage and determination but still need more care for their well-being. Strategies to deal with difficult situations and cultivate well-being must be implemented in medical centers so their employees can experience and practice them. In addition to physical exercise and a balanced diet, behavioral tools such as cultivating positive thoughts and feelings of gratitude, practicing different forms of meditation, Yoga, Tai Chi or other practices similar can also calm the mind and reduce stress.^8–10^
